# What Do the Animal Studies of Stress Resilience Teach Us?

**DOI:** 10.3390/cells10071630

**Published:** 2021-06-29

**Authors:** Marta Dziedzicka-Wasylewska, Joanna Solich, Agata Korlatowicz, Agata Faron-Górecka

**Affiliations:** Maj Institute of Pharmacology Polish Academy of Sciences, Smetna Street 12, 31-343 Kraków, Poland; wasyl@if-pan.krakow.pl (M.D.-W.); solich@if-pan.krakow.pl (J.S.); korlat@if-pan.krakow.pl (A.K.)

**Keywords:** stress, stress resilience, depression, animal model, miRNA

## Abstract

Long-lasting stress factors, both biological and psychological, are commonly accepted as the main cause of depressive disorders. Several animal models, using various stressful stimuli, have been used to find biochemical and molecular alterations that could help us understand the etiopathogenesis of depression. However, recent sophisticated studies indicate that the most frequently used animal models of stress only capture a portion of the molecular features associated with complex human disorders. On the other hand, some of these models generate groups of animals resilient to stress. Studies of the mechanisms of stress resilience bring us closer to understanding the process of adapting to aversive stimuli and the differences between stress-susceptible vs. resilient phenotypes. Especially interesting in this context is the chronic mild stress (CMS) experimental paradigm, most often using rats. Studies using this animal model have revealed that biochemical (e.g., the dopamine D2 receptor) and molecular (e.g., microRNA) alterations are dynamic (i.e., depend on stress duration, 2 vs. 7 weeks) and much more pronounced in stress-resilient than stress-susceptible groups of animals. We strongly suggest that studies aimed at understanding the molecular and biochemical mechanisms of depression must consider these dynamics. A good candidate to serve as a biomarker in such studies might be serum microRNA, since it can be obtained relatively easily from living individuals at various time points.

## 1. Introduction

Depression is a chronic and recurrent psychiatric disorder, characterized by an array of symptoms, the most prominent of which are depressed mood, recurrent thoughts of death and suicide, feelings of worthlessness, social isolation, and anhedonia. A great deal of advanced research in the field has been performed using a modern, sophisticated methodological approach, taking into account various central but also peripheral levels of regulation, i.e., wide genomic alterations [1], the contribution of endocrine processes, including sex differences, and the immunological response associated with depression [2], as well as vascular functions [3], especially with neurovascular adaptations in human depression [4], which were extensively reviewed recently by Dudek et al. [5].

Despite the great efforts to find the biological substrates underlying this complex disease, little research has revealed new therapeutic targets. Access to high-quality brain tissue obtained from well-characterized patients is restricted, but also important are the issues put forward in 2000 by Lewis [6], who, while discussing difficulties in studies of schizophrenia, pointed out that “any given brain alteration observed in schizophrenia can represent three different phenomena—It can be a cause of psychiatric disorder, its consequence or simply a means of the brain to compensate the disorder”. Ten years later, other authors, greatly appreciating Lewis’s statement, decided to revive this discussion in the field of neuroimaging studies of depression [7]. They indicated that “taking a neuroimaging snapshot of the brain at any given point in the time of depression, the image is presumably the result of four interacting components: neural predisposition, depressogenic pathology, changes caused by chronic depression, and compensatory brain mechanisms”. Although important and even crucial, these issues are not widely accepted, which may be one reason for the current situation, where we are still using drugs that act via aminergic neurotransmitter systems, drugs that were discovered by chance in the middle of the 20th century and need lag time for clinical efficacy, a phenomenon we still do not quite understand. Furthermore, a great percentage of patients are unresponsive to any currently available treatment [8,9].

## 2. Animal Models Used in Studies of Depression

Since long-lasting stress factors, both biological and psychological, are commonly accepted as the main cause of depressive disorders [10,11], several animal models have frequently been used to study the impact of stress [12]. However, often the biochemical and molecular alterations observed in these animal models cannot be reliably confirmed in the brains of humans suffering from depressive disorders. Recently, an interesting analysis of transcriptional signatures in humans affected by major depressive disorder (MDD) and in three mouse models of chronic stress disorder was published [13]. The authors compared transcriptional signatures in two brain regions implicated in depression, the medial prefrontal cortex and the nucleus accumbens, in humans with MDD and mice subjected to one of three experimental paradigms: chronic variable stress (CVS), adult social isolation (SI), or, as the paradigm most extensively studied by some laboratories, chronic social defeat stress (CSDS), which additionally provided data on stress-resilient species. Bioinformatics analyses of the available data were complex and extensive; among other findings, differentially expressed genes indicated that each chronic stress model recapitulates a significant subset of gene expression changes observed in brain regions of human MDD, and the specific genes that overlap with human MDD are largely different for each mouse stress model. Three mouse models were also compared with respect to the nature of the stressors used, using physical stressors for CVS (foot shock, tail suspension, and tube restraint) and psychosocial stressors for CSDS and SI (social defeat and social exclusion, respectively). The final conclusion of these comprehensive analyses shows that each of the three mouse models of chronic stress captures a significant portion of the molecular features associated with the broad and complex human disorder.

The most intriguing finding of this study is a significant level of overlap between human MDD and groups of CSDS-resilient animals. Interpreting these results is difficult; some of the human data in this study were obtained from patients treated with antidepressants; therefore, there is a possibility that, as the authors say, “prior antidepressant medication may have triggered the activation of resilient-like molecular programs”. However, only 14 out of 26 samples came from subjects treated with antidepressants. Another interpretation considers the ability of all organisms to cope with stress.

Leaving aside the interpretation of results obtained in very sophisticated studies by Scarpa et al. [13], since it is too hard to comprehend why the brains of depressed patients trigger sets of genes similar to those of resilient mice subjected to CSDS, one has to recall that in recent years the mechanisms of stress resilience have garnered great interest, since understanding the adaptive, allostatic mechanisms that protect individuals against psychopathology can potentially be beneficial to inventing new treatment strategies for more vulnerable individuals [14].

## 3. Stress Resilience

Long-lasting stress factors, both biological and psychological, are the main cause of depressive disorders. One of the core symptoms of depression is anhedonia, which can be defined as a mood disorder involving a person’s inability to feel pleasure. It is now believed that depression is associated with abnormalities in the maintenance of homeostasis and normal neuronal activity in many brain regions [15]. These abnormal conditions reduce an individual’s biological capacity to “resist” and actively cope with stressors. An interesting hypothesis has recently been put forward indicating the epigenetic mechanisms of stress resilience [16]. Still, many people who experience chronic stress maintain optimism, a willingness to act, and overall psychological well-being. Such people are classified in psychological research as resilient to stress [15,17]. Over the past few decades, many interesting basic and clinical studies have been conducted to determine the molecular and physiological determinants of depression [18]. Of these, only a handful of experiments have attempted to elucidate the biochemical mechanisms underlying psychological stress resilience in humans. The question arises as to whether the stress-resilient or stress-susceptible phenotype is constant during an individual’s life. If so, what is the extent of the ability of resilient individuals to resist the negative effects of stress? The answers to these questions are of great importance, highlighting the need for experiments on molecular and biochemical determinants of stress resilience. In this context, an ideal approach for basic research on the stress-resilient phenotype is to use yet another animal model besides the one characterized recently by Scarpa et al. [13], namely, the chronic mild stress (CMS) behavioral paradigm.

## 4. Chronic Mild Stress Model in Studies of Stress Resilience

CMS is an established animal model of depression that meets all validation criteria (construct, face, and predictive validity) [19]. In the CMS procedure, anhedonia, which is one of the two core symptoms of depression (meeting the face validity criterion), is observed in stressed animals. The occurrence of anhedonia in animals experiencing prolonged mild stress can be defined by measuring the level of consumption of a sweet (and pleasant to the animals) 1% sucrose solution. Animals that are under stress and exhibiting anhedonia drink significantly less sucrose solution, reflecting their reduced sensitivity to rewarding stimuli and reduced motivation. The use of clinically approved antidepressants and electroconvulsive therapy in chronically stressed animals leads to the abolition of anhedonia, which leads to increased sucrose drinking (meeting the predictive validity criterion) [20,21]. During the CMS procedure, a series of mild stress stimuli are inflicted in an uncontrolled and unpredictable manner on the animals, which mimics the natural effects of the stressors and prevents the animals from developing habituation (meeting the construct validity criterion).

Besides behavioral alterations, CMS induces various morphological, cellular, and molecular changes in the brain [22,23]. It has also been observed that this model generates a population of animals that are much better at behaviorally coping with the negative effects of stress [24,25,26]. These animals show no change in their level of drinking a sweet sucrose solution even though they are experiencing chronic mild stress. In the classical methodological approach of the CMS model, animals that do not show changes in sucrose drinking while experiencing mild stress are rejected from the experiment as not responding to the model assumptions. This group of animals should be defined as behaviorally stress-resistant (resilient) individuals, which can be a good model for studies leading to the identification of molecular markers of stress resilience. Given the extension of the CMS model assumptions to stress-resilience criteria, it becomes possible to study the biochemical complexity of different types of stress responses under controlled laboratory conditions. Furthermore, by including a group of animals exhibiting behavioral stress resilience in the data analysis, the model itself fulfills an additional validation criterion by mimicking the natural variation in stress responses seen in the human population, in that not all individuals experiencing chronic stress become depressed (additional evidence of face validity).

## 5. Examples of Dynamic Biochemical Alterations in CMS-Resilient Animals

Major neuromodulatory systems, i.e., noradrenergic, serotonin, and dopaminergic systems, undoubtedly play important roles in the pathogenesis of depressive disorders resulting from the experience of long-term stress. Disturbed transmission of these neurotransmitters is observed in both depressed individuals and animal models of depression. This observation has led to the further development of many antidepressants targeting aminergic systems (e.g., reuptake inhibition, antagonism toward autoreceptors). It should be noted that the effects of antidepressant therapy with drugs that regulate serotonergic or noradrenergic transmission occur only after several weeks of use, while drugs that affect the dopaminergic system are marginally used due to the possibility of addiction. Nevertheless, studies indicate the important involvement of dopamine D2 receptors in the mesolimbic system in the pathogenesis of depression and the mechanisms of action of antidepressant drugs [27,28,29], as well as in the generation of the stress-resilient phenotype.

Interesting dynamic alterations at the level of dopamine D2 receptor in the striatum, nucleus accumbens septi, and ventral tegmental area were observed in animals subjected to CMS for shorter (2 weeks) and longer (7 weeks) periods [24]. These changes were strictly dependent on the duration of the stress factors. In the stress-resilient phenotype, a strong decrease in dopamine D2 receptor expression was observed in the mesolimbic system during the first phase (after 2 weeks of CMS), which was not observed in the anhedonic (stress reactive) animals. During prolonged stress (after 5 weeks of CMS), the opposite effect was observed: the level of dopamine D2 receptors in the mesolimbic system of the group of stress-resistant animals did not differ from the level in unstressed animals; moreover, the level of mRNA encoding the dopamine D2 receptor in the ventral tegmental area increased in resistant animals. Such changes were not observed in anhedonic animals. In this group, significant decreases in dopamine D2 receptor expression were observed in all the brain regions examined during prolonged stress. These results demonstrate the existence of an active biochemical mechanism counteracting the effects of stress at the level of the mesolimbic system in resilient animals. In animals exhibiting anhedonic behavior, this biochemical response is delayed and not habituated. If only one of the two time points were studied, no such observation would have been possible. Similarly, Faron–Górecka et al. [25] showed the effects of CMS on plasma prolactin levels and its receptors in the choroid plexus, the alteration of which was significantly dependent on the duration of stress.

Strong changes in dopamine D2 receptor density without changes in the expression level of mRNA encoding this receptor after a 2-week CMS procedure may indicate the involvement of miRNAs as post-transcriptional regulators of gene expression. The first miRNA was discovered in 1993 by Victor Ambros et al. [30] while studying *C. elegans* development. They found that the short (22 nucleotides) RNA molecule negatively regulated the expression of the LIN-14 protein in this nematode. Since then, many families of these small oligonucleotides have been revealed. miRNA molecules are widely expressed in eukaryotes. miRNAs are small (17–24 nucleotides), noncoding RNA transcripts that play an important role in post-transcriptional regulation of many genes. miRNA binds to the 3’UTR region of the targeted mRNA and causes its degradation by recruiting RISC protein complexes. It is worth noting that animal miRNAs exhibit partial complementarity to targeted mRNAs. Therefore, one miRNA can regulate the translation of more than one protein. In the human genome, over 1000 miRNAs have been identified, which may target about 30% of genes. In addition, miRNA oligonucleotides are widely expressed in all animal tissues. These molecules are present in blood plasma/serum and in other biological fluids such as saliva, cerebrospinal fluid, and semen as extracellular nuclease-resistant entities [31].

The origin of extracellular miRNAs is not fully understood. It seems that they can be actively secreted by cells and, in effect, play an informative role between cells and tissues. This hypothesis is explained by the presence of miRNA packed in exosomes, shedding vesicles, and lipoproteins (HDL) in the blood. However, a vast majority of extracellular miRNAs are associated with proteins of the Argonaute (AGO) family, indicating that these oligonucleotides may be released by dead cells [31]. Because of the impressive stability of circulating miRNAs in the bloodstream and often specific tissue origin, extracellular miRNAs may serve as potential noninvasive biomarkers of many diseases, including brain pathology [32].

## 6. MicroRNA in CMS-Resilient Animals

Based on the screening analysis of 380 mature miRNAs by RT-qPCR, we were able to demonstrate that the animals that were behaviorally resilient to CMS had elevated serum miR-16-5p levels during the first week of CMS and in response to prolonged stress, i.e., at weeks 6 and 7 of the procedure [33]. This peripheral change was also observed in the brain; stress-resistant animals showed elevated miR-16-5p expression levels in the ventral tegmental area and concomitant reduced miR-16-5p levels in the prefrontal cortex after 2 weeks of the stress procedure. The changes in both brain structures showed a significant negative correlation: increased miR-16-5p expression in the ventral tegmental area was accompanied by decreased miR-16-5p expression in the prefrontal cortex of animals exhibiting behavioral stress resilience. These changes were not observed in animals exhibiting anhedonia. Therefore, miR-16-5p could potentially be a peripheral biochemical marker of stress response, as changes in its levels were seen in both brain structures and sera of animals exhibiting resistance to long-term stress. However, a correlation analysis between serum miR-16-5p levels and miR-16-5p expression levels in individual brain structures at given time points showed no direct connection between peripheral and central changes in these levels. This may indicate that miR-16-5p is involved in regulating the total natural capacity of the body to cope with stress, which in turn is the sum of its various tissue-specific actions. miR-16-5p was first reported by Baudry et al. [34] as a candidate for adaptive response to fluoxetine. Then, the idea was expanded by the same group [35] to include additional serotonin reuptake inhibitors, and by others [36], who showed that miR-16 in CSF was decreased in depressive patients.

Bioinformatics analysis using, among other approaches, the freely available myMiRsite program, which calculates the probability of interaction of miRNAs with target RNAs, showed that there is a high probability that miR-16 is involved in the regulation of nervous system function and regulates the level of the transporter for serotonin (SERT) [33]. Again, the results suggest that the de facto stress-resilient phenotype is characterized by biochemical changes that are highly dynamic. In response to short-acting stress (CMS of 2 weeks), changes in miRNA, corticosterone, and receptor levels are observed in resilient animals. These changes become somewhat blunted (normalized) during longer-lasting stress. Rapid biochemical or molecular activation occurring in resilient animals during short-term stress reflects the full mobilization of the organism to fight the negative effects. The subsequent blunting of the initiated changes is most likely responsible for protecting the organism from the devastating effects of prolonged stimulation. Since dynamic changes were not observed in animals exhibiting anhedonia, it may be concluded that their ability to counteract the effects of stress actively and dynamically is reduced or delayed.

## 7. MicroRNA as Biomarkers of Stress Resilience

MicroRNAs have garnered attention as potential biomarkers for monitoring the therapeutic response to antidepressants [37]. As mentioned above, the most common treatment method for MDD is antidepressant drugs, which act by regulating brain monoaminergic systems (by inhibiting presynaptic reuptake and/or modulating monoamine receptors). The choice of appropriate therapy is usually based on subjective decisions made by the physician, and it requires time to determine the treatment outcome and define whether the prescribed treatment is effective. As suggested by Belzeaux et al. [37], biomarkers would help to identify individuals with MDD who are more likely to respond to specific antidepressant treatments, which would provide objectivity in treatment decision-making.

The origin of extracellular miRNAs is not fully understood. It seems that they can be actively secreted by cells, and, in effect, they play an informative role between cells and tissues. This hypothesis is explained by the presence of miRNAs packed in exosomes shedding vesicles and lipoproteins in the blood. Because of the impressive stability of circulating miRNAs in the bloodstream, and their often-specific tissue origin, extracellular miRNAs could serve as potential non-invasive biomarkers of many diseases (including cancers) and as potential biomarkers of brain pathologies, including depressive disorders [37,38]. Indeed, there is often no correlation between the serum level of miRNAs and brain miRNA expression [39], but this may be due to the inadequate measurement of their levels in brain tissue. In studying the brain level of miRNAs, we should pay more attention not only to specific brain regions, but also to the cell types that might be sources of given miRNAs.

We are strongly convinced that microRNAs could serve not only as biomarkers of antidepressant response, but also as a measure of stress resilience. Since they are released by cells and circulate in biological fluids such as blood, microRNAs can easily be measured with a diverse range of robust techniques, such as small RNA sequencing, microarrays, and quantitative polymerase chain reaction (PCR). In recent studies, using three genotypes of mice that were shown to display different sensitivity to restraint stress (RS) [40], we identified a set of 25 miRNAs altered by RS [41]. These miRNAs are responsible for regulating mRNAs encoding proteins that are key to the main hypotheses of depression (e.g., monoaminergic receptors and their transporters, neurotrophic factors, neuropeptides and their receptors, glucocorticoid receptors, glutamate and GABA receptors and their transporters, as well as interleukins, TNF, interferon, and their receptors). These findings additionally support the potential of serum miRNA measurement, which could be used to monitor the dynamics of alterations in various stages of the development of stress resilience.

## 8. Final Conclusions

The data described above show examples of the dynamics of alterations (at various levels of regulation) during the time when an organism experiences adverse stimuli. Using CMS as an animal model of depression (and stress resilience), we were able to show that the biochemical alterations observed were not only strongly dependent on the stress duration, but also more pronounced in the resilient group of animals. These data indicate that dealing with stress is more an active and dynamic process, strategy, or lifestyle than a passive, endogenous resistance to stressful influences, and this aspect should be adequately considered in animal studies.

Interestingly, recent behavioral studies using variable stress in mice, although basically aiming at deciphering sex differences in reaction to stress, additionally showed that a shorter duration of 6 days of stress induced stronger effects in females than males, and that both sexes were equally vulnerable to 28 days of variable stress but habituated to 56 days of stress [42]. Moreover, there are few comprehensive papers addressing the molecular and cellular mechanisms underlying different responses to stress depending on sex and age [43,44,45,46]. Generally, papers concentrate on transcriptome profiling in animals subjected to chronic social defeat stress and in human tissue (post-mortem samples from depressive patients). Age- and sex-specific miRNAs have not been studied so far, but they could certainly serve as markers in specific cases. This will be possible if we obtain deeper knowledge on possible alterations in the levels of specific miRNAs depending on sex and age.

Studies aimed at understanding the molecular and biochemical mechanisms of stress resilience, which can bring us closer to an understanding of depressive disorders, must consider these dynamics. Otherwise, none of the most sophisticated and advanced methodologies will provide solutions adequate for more effective treatment of patients.

## Data Availability

No new data were created or analyzed in this study. Data sharing is not applicable to this article.
